# Animal and human food safety of chromium propionate in layer hen diets

**DOI:** 10.1016/j.psj.2026.106893

**Published:** 2026-04-01

**Authors:** Dimitri Malheiros, Jerry W. Spears, Becca Wysocky, Karen E. Lloyd, Kristi Krafka, Ramon D. Malheiros, Kenneth E. Anderson

**Affiliations:** a Prestage Department of Poultry Science, North Carolina State University, Raleigh, NC 27695-7608, USA; b Department of Animal Science, North Carolina State University, Raleigh, NC 27695-7621, USA; c Kemin Agrifoods North America, Inc., Des Moines, IA 50317, USA

**Keywords:** Chromium propionate, Layer hens, Animal safety, Tissue chromium, Egg chromium

## Abstract

The objective of this study was to determine the animal and human food safety of KemTRACE® chromium propionate (**Cr Prop)** when supplemented to layer diets at 1 and 5 times the recommended level. Three-hundred 21-wk-old Hy-Line W-36 layer hens were randomly assigned to treatments consisting of: Cr Prop supplemented to provide 0, 0.20, or 1.0 mg Cr/kg diet. The study was conducted from 21 through 36 wk of age. Eggs were collected during wk 24, 28, 32, and 36 for egg quality measurements. Eggs were collected on d 0, 28, 56, 84, and 112 for Cr analysis. At the end of the study blood was collected from the wing vein for plasma biochemical measurements and samples of liver, breast muscle, kidney, and skin (with adhering fat) were then obtained for Cr analysis. In wk 21-24 ADFI and egg production were lower (*P* = 0.01) in hens receiving 1.0 mg Cr/kg compared to the other treatments. Egg production was not affected by treatment from wk 25 - 36. This suggests an adaptation to the high level of Cr over time. Over the entire 16-wk study, ADFI was greater (*P* = 0.01) for hens receiving 0.20 or 1.0 mg Cr/kg diet than controls and egg production was lower (*P* = 0.01) for hens supplemented with 1.0 mg Cr/kg compared with those in other treatments. Egg yolk color was lower (*P* = 0.03) for hens receiving 1.0 mg Cr. Other egg quality measurements were not affected by treatment. Hens supplemented with 0.20 mg Cr/kg had lower (P ˂ 0.05) plasma cholesterol, chloride, lipemic index, and bile acid concentrations than controls. Supplementation of 1.0 mg Cr/kg increased (P ˂ 0.05) liver Cr relative to the control treatment, but did not affect Cr concentrations in eggs, breast muscle, kidney, or skin with adhering fat. Tissue Cr concentrations were similar in hens receiving 0 or 0.20 mg Cr/kg. Results support the human food and animal safety of Cr Prop supplemention to layer diets for up to 16 wk.

## Introduction

Chromium (**Cr**) functions to potentiate the action of insulin in insulin-sensitive tissues (Spears, 2025). Supplementation of Cr, in the form of KemTRACE® Cr propionate (**Cr Prop**), enhanced insulin sensitivity in broilers (Brooks et al., 2016) and turkeys (Spears et al., 2024a). The U.S. Food and Drug Administration Center for Veterinary Medicine (FDA) approved a food additive petition in 2016, which allowed the use of KemTRACE® Cr Prop as a source of supplemental Cr in broiler diets at a concentration up to 0.20 mg Cr/kg diet. In 2024 FDA approved KemTRACE® Cr Prop supplementation to growing turkeys at a level up to 0.20 mg Cr/kg diet. Chromium Prop supplementation has increased weight gain and reduced feed/gain ratio in broilers (Arif et al., 2019; Van Hoeck et al., 2020, and increased weight gain in turkeys (Spears et al., 2024b)

Several studies have evaluated Cr supplementation of layer diets from Cr picolinate (Cr Pic; Lien et al., 1996; Sahin et al., 2001) or Cr methionine (Cr Met; Mirfendereski and Jahanian, 2015; Torki et al., 2018) and results have been variable. In a 56-d study with layers during the late laying period (60 to 68 weeks), Cr Prop supplementation (0.40 mg/kg) increased laying rate from 65 to 68 weeks (Ma et al., 2014). The present study was conducted to determine: 1) animal safety of KemTRACE® Cr Prop in layer diets based on egg production, egg characteristics, and plasma clinical chemistry variables, and 2) the effect of supplementing layers with Cr Prop on human food safety based on chromium concentrations in eggs and edible tissues. This study was part of a food additive petition to be submitted to FDA requesting approval of Cr Prop supplementation to layer and broiler breeder hen diets at up to 0.20 mg Cr/kg diet.

## Materials and methods

### Ethical statement

The North Carolina State University (NCSU) Animal Care and Use Committee approved (IACUC 19-581) all care, handling, and sampling procedures utilized in this study.

### Rearing birds

Hy-Line W-36 pullets were grown at the North Carolina State University Piedmont Research Station in Salisbury, NC using their standard protocol. At 18 wk of age hens were moved to the North Carolina State University Poultry Educational Unit in Raleigh, NC.

### Experimental design

Three-hundred 21-wk-old Hy-Line W-36 layer hens were selected for use in the study. The study was conducted from July 21 to November 15, 2022. Hens were randomly assigned to dietary treatments based on egg production prior to initiation of the study. The study was conducted from 21 through 36 wk of age. Peak egg production was expected around 28 wk of age. Treatments consisted of: 1) control (no supplemental Cr), 2) 0.20 mg supplemental Cr/kg diet, and 3) 1.0 mg supplemental Cr/kg diet. Supplemental Cr was provided as Cr Prop (KemTRACE® Cr Prop). Each treatment consisted of 10 replicates, and each replicate consisted of 5 consecutive cages, with 2 hens housed per cage. One mg of supplemental Cr per kg diet is five times the level of Cr Prop that will be requested to FDA for use in layer and broiler-breeder diets. The 0.20 mg Cr/kg supplementation level is based on the minimal level of Cr Prop that increased insulin sensitivity in broiler breeder hens. Treatments were color coded and were blinded to all personnel except the Principal Investigators.

### Housing, diets and management

Hens were identified by wing bands and housed two per cage in Big Dutchman layer cages measuring 13″ W x 18″ D x 23.5″ H. The house consisted of 8 rows of cages with each row containing 102 cages. One row of cages consisted of six replicates per treatment, and a second row consisted of four replicates per treatment. The five consecutive cages within a replicate were randomly assigned to treatments. An empty cage was located between treatment replicates to prevent feed contamination. The lighting regimen was 16L:8D. Lighting was provided from 5:00 am to 9:00 pm using 2700 K white LED light bulbs. Cages were set up with waterers and feeding troughs.

Ingredient composition of the basal mash diet is shown in Table 1. Calculated levels of nutrients in the basal diet are shown in Table 2. Analyzed chemical analysis of the control diet is presented in Table 3. The phytase added to the diet provided 750 units/kg diet. The diet was formulated to meet or exceed Hy-Line W-36 specifications (Hy-Line W 36 Commercial Layers, Management Guide) for layer from 21 to 36 wk of age. Chromium Prop was mixed with finely ground corn to provide either 0, 0.20, or 1.0 mg Cr/kg diet, when added at 0.5 % to the diet.

**Table 1:** Ingredient composition of the layer diet.

Ingredient	%
Corn	44.0
Soybean meal (48% CP)	32.0
Wheat middlings	6.1
Corn-Cr premix	0.5
Calcium carbonate	10.8
Mono-dicalcium phosphate	1.5
Soybean oil	3.9
DL-Methionine	0.345
L-lysine	0.026
Sodium Chloride (Salt)	0.35
Choline Chloride	0.10
Mineral Mixture^a^	0.20
Vitamin Mixture^b^	0.10
Selenium premix (0.06% Se)	0.05
Santoquin	0.05
Phytase (Quantum Blue, 5,000 units/gram)	0.015

a Provided per kg of diet: 120 mg Zn (from ZnSO_4_), 120 mg Mn (from MnSO_4_), 80 mg Fe (from FeSO_4_), 10 mg Cu (from CuSO_4_), 2.5 mg I (from CaIO_4_).

b Provided per kg of diet: vitamin A, 13,227 IU; vitamin D, 3,960 IU; vitamin E, 66 IU; vitamin B_12_, 0.04 mg; riboflavin, 13.2 mg; niacin, 110 mg; d-pantothenate, 22 mg; menadione, 4.0 mg; folic acid, 2.2 mg; thiamine, 4.0 mg; pyridoxine, 7.9 mg; d-biotin, 0.25 mg.

**Table 2:** Calculated composition of diet.

Nutrient	
DM, %	90.2
CP, %	19.5
Crude fat, %	6.0
Crude fiber, %	2.9
ME, kcal/kg	2,813
Methionine, %	0.62
Cystine, %	0.29
Lysine, %	1.10
Tryptophan, %	0.26
Threonine, %	0.75
Isoleucine, %	1.01
Histidine, %	0.50
Valine, %	0.97
Leucine, %	1.67
Arginine, %	1.37
Phenylalanine, %	0.97
Ca, %	4.51
Available P, %	0.45

**Table 3:** Chemical analysis of the control diet.

Nutrient	
DM, %	93.1
CP, % DM	22.3
ADF, %DM	2.9
Ca, %DM	4.82
P, %DM	0.79
Mg, %DM	0.25
K, %DM	1.00
Na, %DM	0.18

Feed and water were offered ad libitum. Feed was weighed and added to feeders as needed. Feed consumption was measured per replicate (5 cages) every 4 wk by weighing feed remaining in feeders. Hens were individually weighed on d 0, 28, 56, 84, and 112 of the study. Egg production was measured daily, and egg production data were divided into four 4-wk phases.

Hens were observed daily for general health. Any hens that were removed or died during the study were necropsied at the North Carolina Veterinary Animal Disease Laboratory to determine the cause of death.

### Sampling

Samples of experimental diets were collected weekly from bags of feed and composited by 4-wk period for Cr analysis. Water samples were collected and acidified with 1 N hydrochloric acid (HCL; 50 ml water + 300 µl HCL) weekly for Cr analysis. The control diet was analyzed for chemical components. Twelve eggs in total per replicate were collected during wk 24, 28, 32, and 36 for quality measurements including egg weight, shell strength, shell elasticity, vitelline membrane elasticity, vitelline membrane hardness, albumin height, Haugh unit, yolk color, and shell color. Two eggs per replicate were collected on d 0, 28, 56, 84, and 112 of the study for Cr analysis. Upon arrival at the laboratory on each collection day, eggs collected for Cr analysis were equilibrated at 4°C for 7 d. On d 7, each egg was thoroughly rinsed with deionized water and dried. The egg was then cracked, all egg contents (minus the shell) placed in a disposable plastic cup, and mixed until homogenous (without frothing) using a plastic Deluxe Cordless Mini Mixer (Norpro®, Everett, Washington, USA). The egg mixture was then transferred to individual 50 ml conical tubes and stored at −20°C until processed further for Cr analysis.

At the termination of the study (d 113: wk 36 of age), blood was collected from the wing vein in heparinized tubes from 1 randomly selected hen per replicate. Blood samples were placed on ice until centrifuged at 1,200 x g for 20 min at 10°C. Following blood collection, hens were euthanized via cervical dislocation. Samples of liver, breast muscle, kidney, and fat (skin with adhering fat) were obtained from a similar location in each bird for Cr analysis. Tissue samples were placed in separate plastic bags, sealed, and placed on ice. Upon arrival at the laboratory, tissue samples were frozen at −20°C until processing for Cr determination.

### Analysis

Egg shell strength and elasticity were measured using a texture analyzer (TA.HDplus, Stable Micro Systems, Surry, United Kingdom) equipped with a 250 kg load cell, which measured the total force in grams. Using a more sensitive texture analyzer (TA.XTplus, Stable Micro Systems, Surry, United Kingdom) furnished with a 5 kg load cell and a 1 mm blunt probe, vitelline membrane strength and elasticity were analyzed per the manufacturer’s instructions. Haugh unit and albumen height were analyzed using the TSS QCD System (Technical Service and Supplies, Dunnington, York, United Kingdom). Haugh unit was calculated using the following equation:

HaughUnit=100xLog(albumenheight−−1.7xeggweight+7.6)

Yolk color was determined using the TSS QCD System yolk color scan. Yolk color scan was calibrated using the DSM Yolk Color Fan that determined the color density from lightest to darkest with a range of 1-15 (Vuilleumier, 1969). Shell color was determined based on light reflectivity scope with pure white representing 83.3 % reflectivity, while 0 % represented pure black reflectivity.

Plasma was analyzed for an avian chemistry panel (Roche Diagnostics, Cobas® 6000 c501, Indianapolis, IN) at the Clinical Pathology Laboratory at the NCSU College of Veterinary Medicine. The panel included glucose, uric acid, phosphorus, calcium, total protein, albumen, globulin, cholesterol, alkaline phosphatase (ALP), aspartate aminotransferase (AST), lactate dehydrogenase (LDH), creatine kinase (CK), sodium, potassium, chloride, bicarbonate, anion gap, Na:K ratio, bile acid, icteric index, and lipemic index.

Frozen tissue samples were allowed to thaw at room temperature. Once thawed, samples were removed from plastic bags and thoroughly rinsed with deionized water. Representative samples were then removed from tissues using titanium knives to prevent Cr contamination. Samples were dried and then prepared for Cr analysis by wet ashing with trace metal grade nitric acid using a hot-plate digestion procedure (Lloyd et al., 2010). Frozen egg mixtures were thawed at room temperature. By collection day, the 2 thawed mixed eggs collected per replicate were then pooled together at a 1:1 ratio by weight. Pooled egg samples and feed composite samples were prepared for Cr analysis as described above for tissue samples. Acidified water samples were analyzed for Cr directly without ashing. Chromium concentrations were measured by electrothermal atomic absorption spectrophotometry. The method of standard addition was used for each tissue, pooled egg, feed composite, and water sample to remove matrix effects as described by Lloyd et al. (2010).

Chemical analysis of the control diet was performed at a commercial laboratory (Dairy One Cooperative Inc., Ithaca, NY). Dry matter (AOAC # 930.15), CP (AOAC # 990.03), and ADF (AOAC # 973.18; ANKOM Technology, Macedon, NY)) were determined by AOAC (2023) procedures. Minerals were determined by ICP-OES (Thermo Fisher Scientific Inc., Waltham, MA)

### Statistical analysis

All statistical determinations were analyzed as a completely randomized design using the MIXED procedure of SAS 9.4 (2016). For feed intake, ADG, and egg production, the model consisted of treatment, 4-wk period, and treatment x period. For egg Cr concentrations and egg quality measurements the model consisted of treatment, wk, and treatment x wk. When a significant treatment x wk or treatment x period interaction was observed, data were analyzed by 4-wk period. The model for blood chemistry parameters and tissue Cr concentrations consisted of treatment only. Differences among treatments and 4-wk periods were determined using the PDIFF option of the LSMEANS statement of SAS.

## Results and discussion

### Chromium analysis of diets

Chromium analysis (DM basis) of experimental diets is shown in Table 4. Within treatment, analyzed Cr concentrations across 4 wk periods were similar. Analyzed Cr concentrations in CrProp supplemented treatments were in line with expected values. A large portion of the Cr in the control diet would have been derived from the 1.5 % mono-dicalcium phosphate added to the diet. Chromium concentrations in feed grade phosphate sources have been reported to range from 112 to 163 mg Cr/kg (Spears et al., 2017). A previous study in turkeys suggested that Cr in mono-calcium phosphate was poorly bioavailability (Spears et al., 2024b). Water samples collected weekly averaged 1.12 ng Cr/ml.

**Table 4:** Analyzed chromium concentrations (mg Cr/kg) in experimental dietsa.

	————————- Sampling day ———————–			
Treatment	28	56	84	112
Control	2.84	2.84	2.70	2.86
0.20 mg Cr/kg	3.04	3.06	2.99	3.09
1.0 mg Cr/kg	3.95	3.96	4.03	3.83

a Dry matter basis.

### Mortality

Eight hens were removed from the study and necropsied. Hens removed consisted of two in the control treatment, three in the 0.2 mg Cr/kg group, and three in the 1.0 mg Cr/kg treatment. Necropsy results are presented in Table 5. Most of the hens removed from the study were due to injuries caused by pecking.

**Table 5:** Necropsy results of hens removed from study.

Treatment	Hen / Date a	Necropsy Results
Control		
	031 (7/29/22)	Traumatic injuries to the skin on top of the skull, presumably caused by pecking
	033 (8/3/22)	Laceration of the head (trauma)
0.20 mg Cr/kg		
	39 (7/29/22)	Traumatic injuries to the skin on top of the skull, presumably caused by pecking
	B-216 (8/25/22) 262 (10/18/22)	Only hen in study that did not lay an egg. No diagnosis. The chicken did not have any lesion suggestive of a disease process, and she had a developing ovary and a developed oviduct Cloacal prolapse
1.0 mg Cr/kg		
	243 (7/29/22)	Traumatic injuries to the skin on top of the skull, presumably caused by pecking
	368 (8/2/22)	Open skin wound – trauma most likely caused by pecking from another bird
	052 (8/9/22)	Consistent with pecking or cloacitis

a Hen number and date of necropsy.

### Feed Intake and body weights

Final BW was not affected by treatment (Table 6). Supplementation of Cr Pic to provide 0.10 to 0.80 mg Cr/kg increased final BW in hens exposed to heat or cold stress (Sahin and Sahin, 2001; Sahin et al., 2001) in some studies but not in others (Torki et al., 2014). Average daily gain and ADFI were affected by 4-wk period (*P* = 0.01) and treatment x 4-wk period (*P* = 0.01; Table 6). During wk 21 - 24, ADG was lower (*P* = 0.01) for hens supplemented with 1.0 mg Cr/kg compared to controls and those receiving 0.20 mg Cr/kg diet. Layers supplemented with 1.0 mg Cr/kg had greater (P ˂ 0.05) during wk 25 – 28 than control and those receiving 0.20 mg Cr/kg diet. Overall ADG was not affected by treatment (*P* = 0.76).

**Table 6:** Effect of dietary chromium on body weight and performance of layers.

	Supplemental Cr^1^, mg/kg				P-value
	0	0.20	1.0	SE	Trt
Final BW,g	1609	1623	1613	6.2	0.30
ADFI, g/d^a^					
Overall	98.0^b^	101.3^c^	101.3^c^	0.54	0.01
21 – 24 wk	91.2^b^	92.4^b^	88.1^c^	1.0	0.01
25 – 28 wk	95.7^b^	100.7^c^	98.0^b,c^	1.3	0.04
29 – 32 wk	101.1^b^	104.6^c^	110.2^d^	1.0	0.01
33 – 36 wk	103.9^b^	107.4^c^	108.7^c^	0.9	0.01
ADG, g/d^a^					
Overall	1.59	1.54	1.50	0.08	0.76
21 −24 wk	2.86^b^	2.44^b^	1.56^c^	0.21	0.01
25 – 28 wk	1.33^b^	1.54^b^	2.27^c^	0.17	0.01
29 – 32 wk	1.39	1.49	1.51	0.15	0.84
33 – 36 wk	0.77	0.69	0.67	0.11	0.82

1 KemTRACE Chromium, 0.40% dry (Kemin Agrifoods North America, Des Moines, IA).

a Effect of treatment x 4-wk period (*P* = 0.01).

b,c,d Means in a row not sharing a common superscript letter differ (P ˂ 0.05).

In wk 21 - 24, ADFI was lower in hens receiving 1.0 mg Cr/kg compared to the other treatments (Table 6). Feed intake averaged 3.9 % lower (P ˂ 0.05) for controls compared to hens supplemented with 0.20 mg Cr/kg from 25 – 36 wk. Average ADFI for control hens was 6.4 % lower (P ˂ 0.05) than those receiving 1.0 mg Cr/kg from 29 – 36 wk. Over the entire 16-wk study, ADFI was greater (*P* = 0.01) for hens receiving 0.20 or 1.0 mg Cr/kg diet than controls. In agreement with our finding supplementation of 0.50 or 1.0 mg Cr/kg, from Cr Met increased feed intake of layers from 28 to 38 wk of age (Mirfendereski and Jahanian, 2015). However, Torki et al. (2018) reported that the addition of 1.2 mg Cr/kg, from Cr Met, reduced feed intake in hens exposed to heat stress. Feed intake has generally not been affected in layers by Cr Pic supplementation (Sahin and Sahin, 2001; Sahin et al., 2001).

### Egg production

Egg production was affected by treatment (*P* = 0.01), 4-wk period (*P* = 0.01), and treatment x 4-wk period (*P* = 0.01; Table 7). As expected, egg production was lower (*P* = 0.01) from 21 – 24 wk than other 4-wk periods. Egg production was also less (*P* = 0.01) from wk 33 – 36 than during wk 25 - 32. Layers supplemented with 1.0 mg Cr/kg diet had lower (*P* = 0.01) egg production during wk 21 - 24 than controls or those receiving 0.20 mg Cr/kg (Table 7). The lower egg production in hens supplemented with 1.0 mg Cr/kg was likely due to their lower feed intake during wk 21 - 24. Egg production was not affected by treatment after 21 – 24 wk. This suggests that hens adapted to the 1.0 mg Cr/kg diet after 4 wk.

**Table 7:** Effect of dietary chromium on egg production in layers.

	Supplemental Cr^1^, mg/kg				P-value
	0	0.20	1.0	SE	Trt
Egg production, %^a^					
Overall	96.9^b^	96.8^b^	95.2^c^	0.22	0.01
21 – 24 wk	92.4^b^	91.6^b^	87.2^c^	0.67	0.01
25 −28 wk	98.7	98.8	98.5	0.27	0.75
29 – 32 wk	99.0	99.0	98.4	0.34	0.38
33 – 36 wk	97.5	97.8	97.1	0.42	0.48

1 KemTRACE Chromium, 0.40% dry (Kemin Agrifoods North America, Des Moines, IA).

a Effect of treatment x 4-wk period (*P* = 0.01).

b,c Means in a row not sharing a common superscript letter differ (P ˂ 0.05).

In late-phase laying hens the addition of 0.40 mg Cr/kg, from Cr Prop, increased egg production from 65 – 68 wk, but not from 60 – 64 wk (Ma et al., 2014). Egg production of hens receiving 0.20 or 0.60 mg Cr/kg did not differ significantly from layers receiving the control or 0.40 mg Cr/kg diet. Responses in egg production to other Cr sources have been variable. Egg production has generally not been affected by supplementation of Cr Pic (Lien et al., 1996; Torki et al., 2014) or Cr Met (Torki et al., 2018) in hens housed under thermoneutral conditions. However, Cr supplementation has increased egg production in layers housed under heat or cold stress (Sahin et al., 2001; Sahin and Sahin, 2001) conditions. Heat stress in the present study was minimal. Average monthly high and low temperatures and monthly high and low daily temperatures during the study are presented in Table 8. Temperatures shown are average of temperatures measured daily in the morning and afternoon at both the front and rear of the house. Although average temperatures in July and August exceeded 30°C, low temperatures were within the zone of thermoneutrality. Feed intake and egg production for wk 21 −24 of the study also suggests that hens experienced minimal heat stress. Egg production and feed intake during wk 21 −24 were above Hy-Line performance expectations.

**Table 8:** High and low inside temperatures in the house during the study.

	Average Monthly Temp,°C		Monthly Daily Temp,°C	
Month	Average high	Average low	High^a^	Low^b^
July	33.7	24.9	34.7	24.6
August	30.6	22.8	34.7	17.9
September	27.6	20.0	30.8	15.7
October	23.0	18.2	27.4	14.6
November	23.6	19.2	26.9	15.7

a High temperature observed during the month.

b Low temperature observed during the month.

### Egg quality

Eggshell strength, elasticity, and color, vitelline membrane strength and elasticity, egg weight, albumin height, and Haugh unit were not affected by treatment or treatment x wk (Tables 9). When analyzed across all wk, yolk color was lower (*P* = 0.01) for hens receiving 1.0 mg Cr/kg and tended (*P* = 0.08) to be lower in those supplemented with 0.20 mg Cr/kg compared to controls (Table 9). The 2.2 % decrease in yolk color in hens supplemented with 1.0 mg Cr/kg compared to controls is probably not of practical importance. Yolk color was only reduced (*P* = 0.03) by Cr on wk 24, when data were statistically analyzed by wk. Ma et al. (2014) reported that compared to controls, yolk color was reduced in late-phase layers receiving 0.20 or 0.40 mg Cr/kg, from Cr Prop, but not in hens supplemented with 0.60 mg Cr/kg. Eggs in this study were collected at the end of an 8-wk study. However, Cr Pic supplementation to provide 0.40 or 0.80 mg Cr/kg reduced eggshell breaking strength following 5 wk of supplementation (Lien et al., 1996). All egg quality measurements, except for eggshell elasticity were affected (*P* = 0.01) by wk in the present study.

**Table 9:** Effect of dietary chromium on egg quality measurements.

	Supplemental Cr^1^, mg/kg				P-value
	0	0.2	1.0	SE	Trt
Shell strength, g/mm^2^					
	4727	4661	4759	51	0.39
Shell elasticity, mm					
	0.26	0.25	0.26	0.003	0.43
Vitelline membrane strength, g/mm^2^					
	2.11	2.05	2.03	0.03	0.18
Vitelline membrane elasticity, mm					
	1.63	1.67	1.61	0.04	0.39
Egg weight, g					
	58.8	59.5	59.5	0.29	0.13
Shell color					
	79.4	78.8	79.7	0.54	0.51
Yolk color					
Overall	7.15^a^	7.04^a^	6.99^b^	0.04	0.03
Week 24	7.40^a^	7.12^b^	7.10^b^	0.09	0.03
Week 28	7.30	7.22	7.10	0.08	0.21
Week 32	7.10	7.07	6.92	0.09	0.31
Week 36	6.80	6.77	6.83	0.10	0.90
Albumen height, mm					
	7.88	7.95	8.02	0.07	0.33
Haugh unit					
	88.7	89.0	89.3	0.4	0.62

1 KemTRACE Chromium, 0.40% dry (Kemin Agrifoods North America, Des Moines, IA).

a,b Means in a row not sharing a common superscript letter differ (*P* ˂ 0.05).

### Plasma clinical chemistry variables

Plasma biochemical measurements at the end of the 16-wk study study are presented in Table 10. Sodium concentrations in plasma were higher (*P* < 0.05) in hens receiving 0.2 mg Cr/kg than in those supplemented with 1.0 mg Cr/kg diet. Plasma sodium concentrations in controls did not differ from either Cr treatment. Hens supplemented with 0.20 mg Cr/kg had higher (*P* < 0.05) plasma chloride concentrations than controls and those receiving 1.0 mg Cr/kg diet. Plasma sodium and chloride concentrations in all treatment groups were within the reference range reported for Hy-Line laying hens Sauer et al. (2019).

**Table 10:** Serum clinical chemistry parameters in laying hens.

	Supplemental Cr^1^, mg/kg diet				P-value
	0	0.20	1.0	SE	Trt
Glucose, mg/dL	227.5	230.5	231.8	3.0	0.59
Uric acid, mg/dL	3.92	3.42	3.33	0.29	0.31
Phosphorus, mg/dL	6.05	5.80	6.51	0.36	0.39
Calcium, mg/dL	33.70	31.64	33.70	1.14	0.35
Sodium, mmol/L	145.6a,b	147.5^a^	144.2^b^	0.79	0.02
Potassium, mmol/L	4.59	4.53	4.86	0.17	0.34
Chloride, mmol/L	111.7^a^	113.9^b^	112.0^a^	0.64	0.05
Total protein, g/dL	5.47	5.08	5.34	0.13	0.12
Albumin, g/dL	2.30	2.18	2.23	0.06	0.34
Globulin, g/dL	3.17	2.90	3.11	0.10	0.16
Cholesterol, mg/dL	227.9^a^	161.4^b^	207.6a,b	17.8	0.04
Bicarbonate, mmol/L	19.9	20.1	19.50	0.62	0.78
Anion gap^c^	18.7	18.1	17.6	0.83	0.65
Lipemic index	125.7^a^	70.9^b^	98.1a,b	16.8	0.09
ALP, IU/L	777	797	881	85	0.66
AST, IU/L	176.5	147.1	151.0	14.0	0.29
LD, IU/L	264.5^a^	170.9^b^	253.4^a^	28.0	0.05
CK, IU/L	711	639	865	155.8	0.58
Icteric index	1.11^a^	0.50^b^	0.80a,b	0.14	0.02
Bile acid	74.2^a^	52.8^b^	69.6a,b	0.38	0.06

1 KemTRACE Chromium, 0.40% dry (Kemin Agrifoods North America, Des Moines, IA).

a,b Means in a row not sharing a common superscript letter differ (*P* < 0.05).

c Sodium + potassium – (chloride + bicarbonate).

Plasma cholesterol concentrations were lower (*P* < 0.05) in hens supplemented with 0.20 mg Cr/kg compared to controls (Table 10). Cholesterol concentrations did not differ among control hens and those receiving 1.0 mg Cr/kg diet. Supplementation of 0.20 to 0.60 mg Cr/kg, from Cr Prop, for 8 wk did not affect plasma cholesterol concentrations in late-phase laying hens (Ma et al., 2014). However, the addition of 0.20 mg Cr/kg, from Cr Prop, reduced serum cholesterol concentrations in broiler breeder hens (Souza et al., 2021). Supplementation of 0.20 or 0.40 Cr/kg, from Cr Pic, reduced serum cholesterol concentrations in heat-stressed hens (Torki et al., 2014). Chromium supplementation at higher levels (0.80 or 1.0 mg Cr/kg) from Cr Pic (Lien et al., 1996) or Cr Met (Mirfendereski and Jahanian, 2015) has reduced plasma or serum cholesterol concentrations in hens under thermoneutral conditions.

Lipemic index and icteric index were also lower (*P* < 0.05) in plasma of hens supplemented with 0.2 mg Cr/kg compared with controls (Table 9). The biological significance of these changes is unclear. Lipemic index measures the lipid content of plasma (Kroll, 2004). Lipemic is caused by large lipoproteins such as chylomicrons or VLDL (very-low-density lipoprotein). The icterus index estimates the amount of bilirubin in the blood. Plasma bilirubin has been used as a measure of liver function (Cornelius, 1989). Plasma lactate dehydrogenase (LDH) activity was also lower (*P* < 0.05) in hens receiving 0.20 mg Cr/kg compared to controls and those supplemented with 1.0 mg Cr/kg diet. Chromium Pic supplementation to provide 0.20 mg Cr/kg also reduced plasma LDH activity in laying hens (Sahin et al., 2018). The lower plasma LDH activity in hens supplemented with 0.20 mg Cr/kg is likely not of biological significance. Elevated plasma LDH would indicate tissue damage. Values for LDH in the present study were similar to previously reported plasma LDH activities in layers (Zhang et al., 2008) and would not be considered elevated. Other plasma clinical chemistry variables measured were not affected by treatment.

### Chromium concentrations in eggs and tissues

Egg Cr concentrations are presented in Table 11. When statistically analyzed by d of the study, supplementation of 0.20 or 1.0 mg Cr/kg diet did not increase egg Cr concentrations on d 0, 28, 56, 84, or 112 compared to controls. When analyzed across d, egg Cr concentrations also were not affected by treatment. Chromium Prop addition at 0.20 or 0.60 mg/kg also did not affect egg Cr concentrations in late-phase layers (Ma et al., 2014). Egg Cr concentrations were affected by day (*P* = 0.01). Chromium concentrations in eggs collected on d 84 and 112 were higher (*P* < 0.05) than those collected on d 0 and 56. Egg Cr concentrations on d 28 did not differ from the other collection days.

**Table 11:** Effect of supplemental chromium on egg chromium concentration in layer hens.

	Supplemental Cr^1^, mg/kg				P-value
	0	0.2	1.0	SE	Trt
	———— ng Cr/g^a^ ————				
Overall^b^	3.56	3.38	3.64	0.22	0.71
Day 0	3.33	2.15	3.47	0.41	0.06
Day 28	3.35	3.81	3.36	0.47	0.73
Day 56	3.10	2.16	3.81	0.50	0.09
Day 84	3.77	4.19	4.16	0.54	0.83
Day 112	4.27	4.60	3.39	0.60	0.36

1 KemTRACE Chromium, 0.40% dry (Kemin Agrifoods North America, Des Moines, IA).

a Expressed on a wet weight basis.

b Effect of day (*P* = 0.01).

Chromium concentrations in breast muscle, skin with adhering fat, and kidney were not affected by supplemental Cr (Table 12). Breast muscle and skin with adhering fat were also not affected in broilers supplemented with 0.40 or 2.0 mg Cr/kg diet, from Cr Prop (Spears et al., 2019). Kidney Cr concentrations were increased by Cr Prop supplementation in lactating dairy cows (Lloyd et al., 2010) but not in turkeys (Spears et al., 2024c). Layers supplemented with 1.0 mg Cr/kg diet had greater (*P* = 0.01) liver Cr concentrations than controls. However, liver Cr in layers supplemented with 0.20 mg Cr/kg did not differ (*P* = 0.36) from controls. Supplementation of Cr Prop to provide 2.0 mg Cr/kg diet also increased liver Cr concentrations in broiler, while liver Cr did not differ between controls and those receiving 0.40 mg Cr/kg (Spears et al., 2019).

**Table 12:** Effect of supplemental chromium on tissue chromium concentrations in layer hens.

	Supplemental Cr^1^, mg/kg				P-value
	0	0.2	1.0	SE	Trt
	——— ng Cr/g ——–				
Breast muscle^a^	32.3	31.8	40.6	5.7	0.48
Skin + Fat^b^	26.5	31.4	33.2	4.7	0.61
Liver^a^	22.7^c^	29.9c,d	43.4^d^	5.3	0.04
Kidney^a^	34.6	27.7	31.1	4.3	0.54

1 KemTRACE Chromium, 0.40% dry (Kemin Agrifoods North America, Des Moines, IA).

a Dry matter basis.

b Air dried value.

c,d Means in a row not sharing a common superscript letter differ (P ˂ 0.05).

## Conclusions

The present study indicates that Cr Prop is safe when supplemented to layer diets for up to 16 wk at 5 x the recommended concentration (0.20 mg Cr/kg). During wk 21 – 24 of the study supplementation with 1.0 mg Cr/kg slightly reduced feed intake and egg production. Egg production was not affected by the high concentration of Cr Prop after 24 wk . Chromium Prop did not affect egg quality or hen mortality, and had minimal effects on plasma chemistry measurements. Although hen mortality was not affected by treatment, a possible limitation of this safety study is that histopathological data and organ weights were not measured. The highest concentration of Cr Prop (1.0 mg Cr/kg) supplemented in the present study was well below the maximum tolerable level for poultry that has been estimated at 500 mg Cr/kg diet (NRC, 2005). Supplementation of Cr Prop did not increase Cr concentrations in eggs, breast muscle, and skin with adhering fat, the major layer products consumed by humans in the U.S. Supplementation of 1.0 mg Cr/kg diet increased liver Cr concentrations by roughly two-fold compared to the control treatment. Consumption of chicken liver by most humans would be extremely low. Assuming a maximum intake of 100 g (wet weight) of liver, consumption of liver from layers supplemented with 1.0 mg Cr/kg diet would increase Cr intake by 2.07 µg/day. The adequate intake of Cr by humans has been estimated at 25 µg for adult females and 35 µg for adult males (National Academies, 2001). Therefore, supplementation of 1.0 mg Cr/kg diet, from Cr Prop to layers for up to 16 wk would have minimal effect on the total Cr intake by humans.

## CRediT authorship contribution statement

**Dimitri Malheiros:** Writing – original draft, Methodology, Investigation, Data curation. **Jerry W. Spears:** Writing – review & editing, Investigation, Conceptualization. **Becca Wysocky:** Writing – review & editing, Methodology, Investigation, Formal analysis, Data curation. **Karen E. Lloyd:** Writing – review & editing, Validation, Methodology, Formal analysis, Data curation. **Kristi Krafka:** Writing – review & editing, Funding acquisition, Conceptualization. **Ramon D. Malheiros:** Writing – review & editing, Investigation, Data curation. **Kenneth E. Anderson:** Writing – review & editing, Project administration, Investigation.

## Disclosures

Kristi Krafka is an employee of Kemin Agrifoods North America, Inc. The other authors declare that they have no conflict of interest in this study.
